# Meet the Editorial Board Member

**DOI:** 10.2174/1570159X2214240709123555

**Published:** 2024-07-09

**Authors:** David J. Calkins

**Affiliations:** 1 Department of Ophthalmology and Visual Sciences,Vanderbilt University Medical Center, Nashville, TN, USA

   Dr. Calkins holds a doctorate (Ph.D.) in Neuroscience from the University of Pennsylvania, Philadelpha. He is currently Professor of Ophthalmology & Visual Sciences and Pharmacology in the Vanderbilt University School of Medicine and Director of the Vanderbilt Vision Research Center. He is also Vice Chair and Director for Research of the Vanderbilt Eye Institute. He has published more over 70 primary research articles, reviews and chapters and serves on the editorial board for several journals.

## References

[1] Wareham L.K., Liddelow S.A., Temple S., Benowitz L.I., Di Polo A., Wellington C., Goldberg J.L., He Z., Duan X., Bu G., Davis A.A., Shekhar K., Torre A., Chan D.C., Canto-Soler M.V., Flanagan J.G., Subramanian P., Rossi S., Brunner T., Bovenkamp D.E., Calkins D.J. (2022). Solving neurodegeneration: common mechanisms and strategies for new treatments.. Mol. Neurodegener..

[2] Calkins D.J. (2021). Adaptive responses to neurodegenerative stress in glaucoma.. Prog. Retin. Eye Res..

[3] Baratta R.O., Schlumpf E., Buono B.J.D., DeLorey S., Calkins D.J. (2022). Corneal collagen as a potential therapeutic target in dry eye disease.. Surv. Ophthalmol..

[4] Lambert W.S., Carlson B.J., Ghose P., Vest V.D., Yao V., Calkins D.J. (2019). Towards a microbead occlusion model of glaucoma for a non-human primate.. Sci. Rep..

[5] Risner M.L., Pasini S., McGrady N.R., Calkins D.J. (2022). Bax contributes to retinal ganglion cell dendritic degeneration during glaucoma.. Mol. Neurobiol..

[6] Taiel M., Sahel J.A., Calkins D.J. (2023). Ocular stress enhances contralateral transfer of lenadogene nolparvovec gene therapy through astrocyte networks.. Mol. Ther..

[7] McGrady N.R., Boal A.M., Risner M.L., Cooper M.L., Pasini S., Lambert W.S., D’Alessandro K.B., Yao V., Risner M.L., Calkins D.J. (2020). Redistribution of metabolic resources through astrocyte networks mitigates neurodegenerative stress.. Proc. Natl. Acad. Sci. U.S.A.

